# The Prevention of Phimosis

**Published:** 1902-02

**Authors:** 


### The Prevention of Phimosis.

In the *Pennsylvania Medical Journal,* F. Le Moyne suggests the following method of preventing phimosis: "One of the early attentions to the male infant should be the gentle and patient retraction of the prepuce and cleansing and anointing of the exposed parts. This should be done as often as is necessary to prevent the accumulation of secretion. The nurse, parent or the child himself, when old enough, may be instructed to assist in the treatment. There will be less danger of its encouraging bad habits than the irritation which is so liable to result from neglect." If these precautions were taken, circumcision would not be so often necessary. R.

